# A new species of *Neohagenulus* Traver, 1938 from Hispaniola (Ephemeroptera, Leptophlebiidae, Hagenulinae, Hagenulini)

**DOI:** 10.3897/zookeys.1070.73484

**Published:** 2021-11-10

**Authors:** Michel Sartori

**Affiliations:** 1 Museum of Zoology, Palais de Rumine, Place Riponne 6, CH-1014,Lausanne, Switzerland Museum of Zoology Lausanne Switzerland; 2 Department of Ecology and Evolution, Biophore, University of Lausanne, CH-1015, Lausanne, Switzerland University of Lausanne Lausanne Switzerland

**Keywords:** *Borinquena*, *Careospina*, *Hagenulus*, morphology, new species, nymph, sexual dimorphism

## Abstract

Here, I report a new species of the genus *Neohagenulus* Traver, 1938 from the Dominican Republic. The genus was believed to be endemic to Puerto Rico until now. *Neohagenulushodeceki***sp. nov.** is described at the nymphal stage. Some discussion on the tribe Hagenulini is also provided.

## Introduction

Nine genera of the family Leptophlebiidae have been found on four islands of the Greater Antilles, Cuba, Hispaniola, Jamaica and Puerto Rico (Naranjo Lopez and Peters 2016) and some of them are sometimes considered to be subgenera (Kluge 1994). Except for *Farrodes* Peters, 1971, *Hagenulopsis* Ulmer, 1920 and *Hagenulus* Eaton, 1882, which include species distributed in the continental Americas, all other genera are endemic to the Greater Antilles. These are *Borinquena* Traver, 1938 with two species in Puerto Rico and one in Cuba, *Careospina* Peters, 1971 with four species in Cuba and one in Hispaniola, *Neohagenulus* Traver, 1938 with three species in Puerto Rico and *Poecilophlebia* Kluge, 1994, *Traverina* Peters, 1971 and *Turquinophlebia* Kluge, 1994, each with one, two and one species endemic to Cuba, respectively (Naranjo Lopez et al. 2019).

Hispaniola is one of the less-studied for mayflies of the four islands. Two leptophlebiid species are currently known, *Hagenuluseatoni* Banks, 1924 and *Careospinaannulata* Peters, 1971. The former is only known by the type series, which consists of 11 pinned females collected in 1912 at Diquini, Haiti and the latter is known only by the holotype, a pinned male imago collected in 1934 from Mont La Hotte, Haiti. Kluge (1994) considered the systematic position of *C.annulata* as unclear, pending the description of the nymph to fix its status.

Here, I report the presence of the genus *Neohagenulus* on Hispaniola, from the Dominican Republic, based on nymphs which are described as a new species. This is the first species of this genus known outside of Puerto Rico.

## Material and methods

The specimens have been collected in the Dominican Republic during a field trip in summer 2021.

Nymphs were preserved in 100% ethanol. Nymphal habitus were photographed using a Canon EOS 6D camera and the Visionary Digital Passport imaging system (formerly available and distributed by Dun Inc., Virginia) and processed with Adobe Photoshop Lightroom and Helicon Focus version 5.3.

Two nymphs were dissected in Cellosolve (2-Ethoxyethanol) with subsequent embedding in Euparal medium and mounting on slides. Fore- and hind wingpads of a submature female nymph were dissected and subimaginal wings examined. Microscopic pictures were taken using an Olympus BX51 microscope coupled with an Olympus SC50 camera; photographs were enhanced with Olympus Stream Basic 2.3.2 stacking software and Adobe Photoshop version 21.2.2.

The material is deposited in the collections of the Museum of zoology, Lausanne (MZL) and the Museo National de Historia Natural "Prof. Eugenio de Jesús Marcano", Santo Domingo, Dominican Republic (MNHNSD).

## Results

### Neohagenulus hodeceki sp. nov.

Taxon classificationAnimaliaEphemeropteraLeptophlebiidae

D29523E8-62E8-5AD6-8AB1-E22F8950AB8D

http://zoobank.org/FDBF727E-87FD-4120-9E62-BDBCECBA8659

#### Material.

***Holotype***. Dominican Republic male nymph in ethanol, La Vega Province, Valle Nuevo National Park, 18°52'01"N, 70°34'44"W, 12 Jul. 2021, ca 900 m a.s.l., J. Hodeček leg. (GBIFCH00834690) [MZL] ***Paratypes***. 5 nymphs in ethanol [MNHNSD] (MNHNSD 11.05 – MNHNSD 11.09), 12 nymphs in ethanol (GBIFCH00834691), 2 female nymphs on slide (GBIFCH00604114-GBIFCH00604115), same data as holotype. [MZL]

#### Other material.

Dominican Republic 1 nymph, La Vega Province, Armando Bermúdez National Park, 19°04'02"N 70°51'50.7"W, ca 1100 m a.s.l., 15 Jul. 2021, J. Hodeček leg. (GBIFCH00834694) [MZL]

#### Etymology.

The new species is named after its collector, Dr Jiří Hodeček (CHUV, Lausanne), forensic entomologist.

#### Description.

***Nymph*** (not mature): body length up to 7 mm, cerci slightly longer than body length, paracercus longer than cerci.

**Figure 1.**
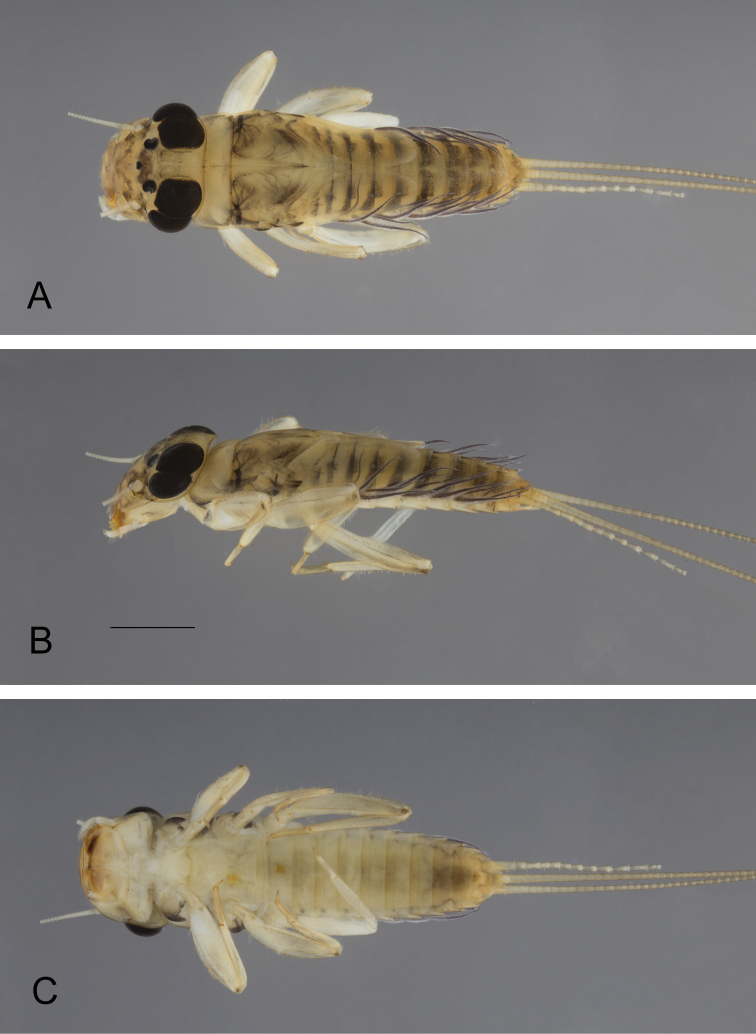
*Neohagenulushodeceki* sp. nov., nymphal habitus **A** dorsal view **B** lateral view **C** ventral view. Scale bar: 1 mm.

***Coloration.*** Cuticular coloration evenly light brown on whole body; hypodermal coloration as in Fig. 1: head washed with grey, darker between ocelli, convex band between antennae, upper portion of male eyes black; prothorax greyish laterally and on posterior margin, mesothorax with blackish tracheation laterally, with maculae posteriorly; legs light brown, apex of femora with blackish dots, tarsi darker than tibiae; abdominal tergites I–V light brown with posterior black band larger laterally and with two antero-submedian maculae, tergites VI–VIII greyish brown, posterior margin and sagittal line light brown, tergite IX paler than previous ones, tergite X medium brown; sternites uniformly light brown, last two darker; cerci uniformly light brown.

**Figure 2.**
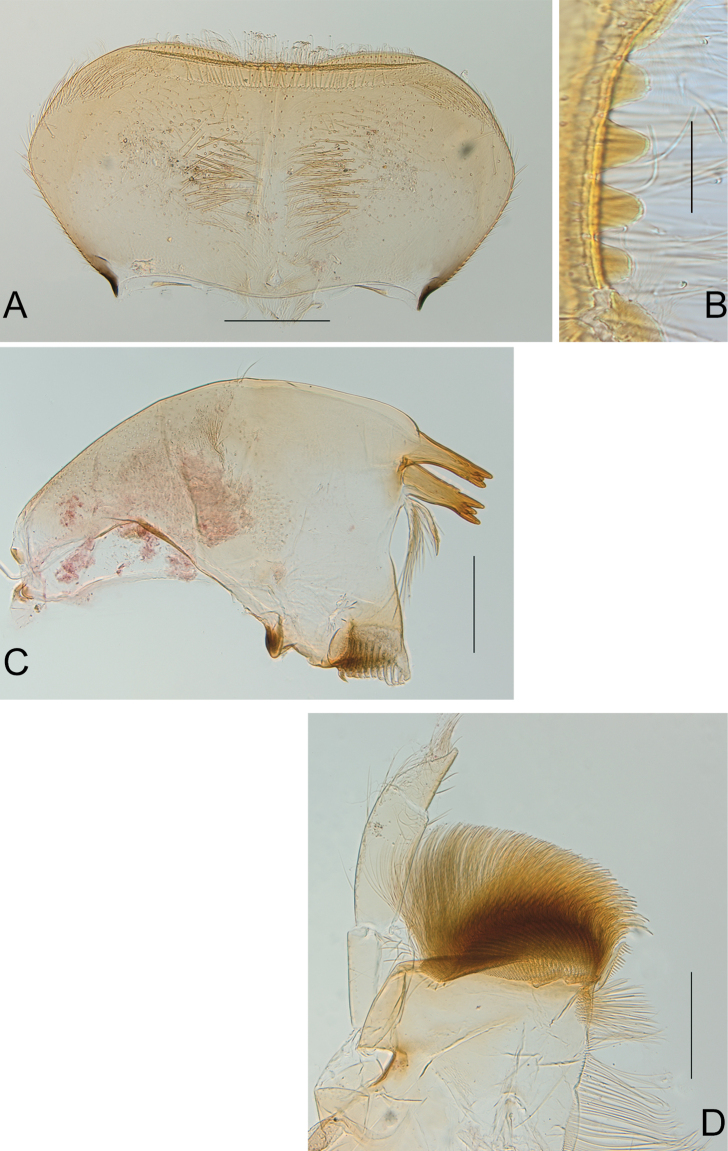
*Neohagenulushodeceki* sp. nov. mouthparts **A** labrum in dorsal view **B** antero-median emargination of the labrum **C** left mandible **D** maxilla. Scale bars: 0.2 mm (**A**, **C**, **D**); 0.05 mm (**B**).

**Figure 3.**
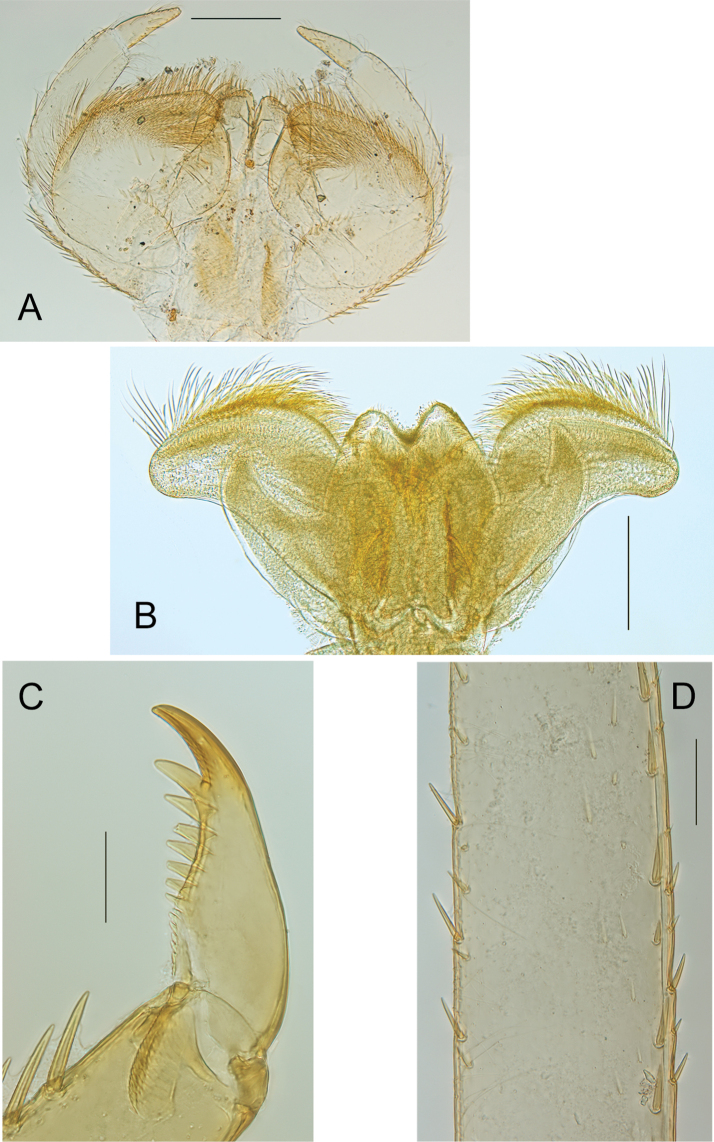
*Neohagenulushodeceki* sp. nov. mouthparts and legs **A** labium **B** hypopharynx **C** claw **D** detail of hind tibia. Scale bars: 0.2 mm (**A–C**); 0.1 mm (**D**)

***Mouthparts.*** Labrum (Fig. 2A) larger than clypeus, about two times broader than long, dorsally with two rows of long and thin setae, proximal row very close to distal margin, tuft of long setae laterally, more abundant at anterolateral corner, ventrally with two submedian fields of long and stout setae, antero-median emargination smooth, with four equally sized denticles (Fig. 2B). Mandibles outer margin regularly convex, with tuft of small and thin setae in middle, with outer and inner incisors composed of three teeth, outer margins slightly serrated, prostheca with stout and long process and well-developed tuft of thin setae (Fig. 2C). Maxillary palp three-segmented (Fig. 2D), second segment ca 1.25× length of segment 1, segment 3 conical, outer margin concave near apex, ca 1.5× longer than wide and 0.50‒0.60× length of segment two, crown of the galea-lacinia with subapical setae arranged in two rows of 8‒9 laterally and 11‒12 centrally. Hypopharynx with lingua convex, with deep incision distally, lateral processes well developed, but shorter than lingua, slightly curved inwards; superlinguae laterally expanded, distal margin covered with long setae up to the tip (Fig. 3B). Labium (Fig. 3A) with narrow glossae, with stout setae at apex, paraglossae rhomboid, outer margin almost straight, with numerous long setae laterally and apically; labial palp three-segmented, first and second segments subequal in length, or segment two slightly longer, third segment ca 0.35‒0.40× length of segment two, 2.5‒2.6× longer than wide.

**Figure 4.**
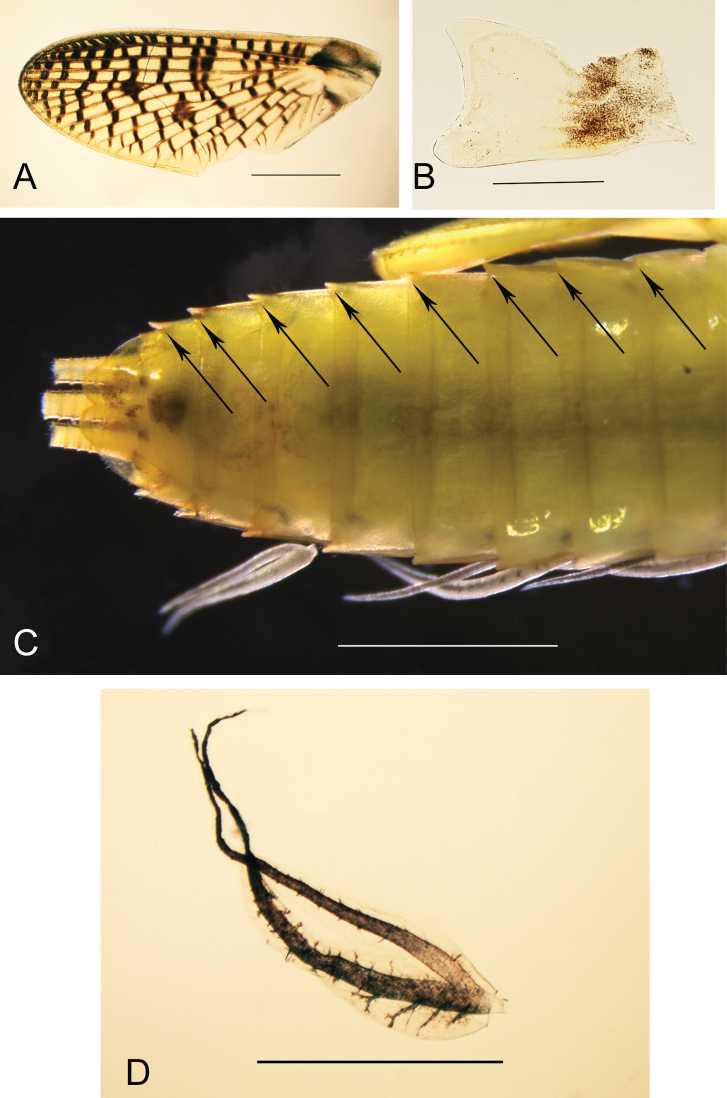
*Neohagenulushodeceki* sp. nov. thorax and abdomen. **A** female fore wingpad **B** female hind wingpad (same specimen) **C** abdomen in ventral view (arrows indicate posterolateral projections on segments II‒IX) **D** gill IV. Scale bars: 0.5 mm (**A**, **D**), 0.2 mm (**B**), 1 mm (**C**).

***Thorax.*** Forelegs with femora ca 2.5× longer than wide, outer margin with row of very long and thin setae, together with apical and subapical rows of long, stout and pointed setae, inner margin with subapical row of long and stout setae (shorter than those on the outer margin), with small stout setae in proximal part, dorsal surface with scattered small to medium-sized stout setae; tibiae shorter than femora, with long and thin setae on outer margin, inner margin with several rows of stout small to medium-sized setae; tarsi only with thin setae, claw moderately hooked, with a row of 12‒14 teeth, increasing in size distally (Fig. 3C). Midlegs similar to forelegs, except inner margin of tibiae with fewer stout setae. Hindlegs with femora almost 3× longer than wide, similar to forelegs in ornamentation except outer margin with shorter and less numerous thin setae; hind tibia shorter than femora, with outer margin covered with long and thin setae, together with short and medium-sized stout and pointed setae, inner margin with marginal row of short and submarginal row of longer stout setae (Fig. 3D). Fore wingpad markedly different between male and female nymphs: in males, evenly brown with veins hardly visible (Fig. 1A), in females with longitudinal veins well marked, crossveins flanked with dark brown bands (Fig. 4A); in both sexes, base of wingpads tinted with brown in costal, subcostal and anal fields. Hind wingpad very small, tinted with greyish brown at base, with costal process large and slightly pointed, almost as long as the rounded apex of wing (Fig. 4B).

***Abdomen***. Posterior margin of tergites I‒III smooth, of segments IV‒VIII with small needle-like denticles, slightly increasing in size posteriorly, tergites IX and X with triangular denticles; posterior margin of sternite IX concave in the middle (Fig. 4C); posterolateral projections on abdominal segments II‒IX, increasing in size posteriorly (Fig. 4C); gills present on segments I‒VII alike, each gill deeply forked almost at base (Fig. 4D) with purplish longitudinal and lateral tracheations, size of gills in decreasing order: II=V>III>IV=VI>I>VII. Cerci with whorls of small setae at the end of each segment.

Male imago, female imago, eggs unknown.

## Discussion

The new species is attributed to the genus *Neohagenulus* mainly based on the posterolateral projections present on abdominal segments II‒IX. In the redescription proposed by Peters (1971), these projections are present on segments III‒IX for Puerto Rican species; this is the main character to separate nymphs of *Neohagenulus* from those of *Careospina*, where these projections are located on segments (V) VI‒IX. Another closely related (sub)genus is *Borinquena*, as defined by Kluge (1994), which possesses an elongated third segment of labial palp, longer than half the length of second segment, which is not the case in *N.hodeceki* (about one-third the length of second segment).

Another consideration for placement of the new species in the genus *Neohagenulus* is the size of the costal projection on the hind wingpad, suggesting that the hind wing of this species possesses a well-developed and long process. In *Careospina*, the costal process is present and normally developed (Peters 1971: fig 29), whereas it is hypertrophied in *Neohagenulus* (Peters 1971: figs 34‒42), reaching the apex of the wing. This character can also be found in *Borinquena* (Kluge 1994: fig 53). All in all, morphological differences between nymphs of *Borinquena*, *Careospina* and *Neohagenulus* are tiny; in *N.hodeceki*, the length of the second segment of the maxillary palp is about 1.25× the length of the first segment, a character which matches the diagnosis of *Careospina* rather than that of *Neohagenulus*, where both segments are of equal size. These three genera seem easier to separate at the male imaginal stage, based on the shape of the hind wing, forceps and penes (Peters 1971; Kluge 1994).

Despite of this, *N.hodeceki* can be separated from species of *Careospina* (all known from Cuba) and *Borinquena* (Puerto Rico and Cuba) at the nymphal stage by the number of posterolateral projections on the abdomen and from species of *Neohagenulus* (all known from Puerto Rico), by the size of the second segment of the maxillary palp.

A remarkable character is the difference in the fore wingpad coloration between male and female nymphs; this dimorphism is not reported within *Neohagenulus* or *Careospina* species, although females often have costal and subcostal fields of forewing more tinted than in males. This difference is nevertheless testified for the species *Hagenulusjamaicensis* Peters, 1971 where male forewing is hyaline except some crossveins “surrounded with brown clouds” at the tip of the wing, whereas the female forewing exhibits almost all crossveins “surrounded with dark brown clouds” (Peters 1971: figs 58, 61).

Among the tribe Hagenulini (sensu Monjardim et al. 2020) and according to Kluge (1994), *Hagenulus* sensu lato, or Hagenulus/fg2, is composed of the following taxa: *Borinquena*, *Careospina*, *Hagenulopsis*, *Hagenuluss.s.*, *Neohagenulus*, *Poecilophlebia* and *Turquinophlebia*, which form a monophyletic lineage. The genus *Borinquena* is reported from Hispaniola only by fossil records from Dominican amber, together with the extinct genus *Hagenulites* Staniczek, 2003 (Staniczek 2003; Staniczek et al. 2017).

The fact that the genus *Borinquena* is known from Puerto Rico and Cuba by five extant species and from Hispaniola by four extinct species indicates a larger distribution of the genus in the Miocene. Due to the lack of prospection on Hispaniola, it is therefore possible that the genus is still present but overlooked. In any case, a better understanding of the diversity of the tribe Hagenulini on Hispaniola is required before any biogeographical or phylogeographic attempt.

*Neohagenulushodeceki* is the third extant leptophlebiid species reported from Hispaniola, the first one for almost a century and the first one known at the nymphal stage. Although I fully agree that the knowledge of the imaginal stages is of prime importance, I think it is noteworthy to formally mention and describe this taxon due to the scarcity of data on the leptophlebiids of Hispaniola. Considering the diversity reported for fishes (Rodriguez-Silva and Schlupp 2021) and caddisflies (Flint and Sikora 2004) for instance, we might deduct that mayfly diversity in Hispaniola is greatly underestimated.

## Supplementary Material

XML Treatment for Neohagenulus hodeceki
